# Development of a prognostic index and screening of potential biomarkers based on immunogenomic landscape analysis of colorectal cancer

**DOI:** 10.18632/aging.102979

**Published:** 2020-03-31

**Authors:** Kang Lin, Jun Huang, Hongliang Luo, Chen Luo, Xiaojian Zhu, Fanqin Bu, Han Xiao, Li Xiao, Zhengming Zhu

**Affiliations:** 1 Department of Gastrointestinal Surgery, The Second Affiliated Hospital of Nanchang University, Nanchang 330006, Jiangxi, P.R. China

**Keywords:** colorectal cancer, immunogenomic landscape, the cancer genome atlas, prognostic index, personalized medicine

## Abstract

Background: Colorectal cancer (CRC) accounts for the highest fatality rate among all malignant tumors. Immunotherapy has shown great promise in management of many malignant tumors, necessitating the need to explore its role in CRC.

Results: Our analysis revealed a total of 71 differentially expressed IRGs, that were associated with prognosis of CRC patients. Ten IRGs (FABP4, IGKV1-33, IGKV2D-40, IGLV6-57, NGF, RETNLB, UCN, VIP, NGFR, and OXTR) showed high prognostic performance in predicting CRC outcomes, and were further associated with tumor burden, metastasis, tumor TNM stage, gender, age, and pathological stage. Interestingly, the IRG-based prognostic index (IRGPI) reflected infiltration of multiple immune cell types.

Conclusions: This model provides an effective approach for stratification and characterization of patients using IRG-based immunolabeling tools to monitor prognosis of CRC.

Methods: We performed a comprehensive analysis of expression profiles for immune-related genes (IRGs) and overall survival time in 437 CRC patients from the TCGA database. We employed computational algorithms and Cox regression analysis to estimate the relationship between differentially expressed IRGs and survival rates in CRC patients. Furthermore, we investigated the mechanisms of action of the IRGs involved in CRC, and established a novel prognostic index based on multivariate Cox models.

## INTRODUCTION

Colorectal cancer is a heterogeneous disease that results from accumulation of mutations over many years under the influence environmental and genetic factors [1]. CRC is ranked in the top three most fatal cancers in the United States, resulting in high rate cancer-related deaths [2]. Currently, the most effective therapies for CRC include laparoscopic surgery for primary tumors (although highly aggressive surgeries are required in advanced cases), combined with radiation therapy and palliative chemotherapy. However, the therapeutic effect of these drugs for advanced and metastatic tumors remains suboptimal [3]. Over the past decade, immunotherapy-based drugs have been extensively explored for development of cancer treatments. Immunotherapy has, therefore, become an effective therapy for several cancers, such as CRC [4, 5] revolutionizing its treatment to a great extent. Some limitations of immunotherapy can be circumvented by combining immune checkpoint inhibition using epigenetic therapy. Drugs that target programmed cell death, including protein 1(PD1) and Cytotoxic T-lymphocyte-associated protein 4 (CTLA-4), have been effective in treatment of not only melanoma, but also other forms of lung cancers and Hodgkin lymphoma. For instance, nivolumab and pembrolizumab are marketed as effective drugs for CRC patients. Based on this, it is evident that immunotherapeutic approaches that can control development of CRC have shown potential for long-term and durable remission of the disease [6–12].

In this study, we sought to determine the clinical role of immunity genes as tools for classifying prognosis of CRC patients and the possibility of such genes to serve as CRC treatments. We performed bioinformatics analysis to reveal expression profiles of these genes in CRC across various clinical traits. We employed multiple computational methods to systematically assess the relationship between IRG and survival time of CRC patients and analyzed the relationship between our constructed prognostic model with various immune cells. Taken together, the findings herein are expected to advance application of the knowledge on immunotherapy and personalized CRC treatment.

## RESULTS

### Summary of the results

Differential gene expression analysis revealed 6524 differentially expressed genes (DEGs), 484 differentially expressed IRGs and 70 differentially expressed transcription factors (TFs). After gene enrichment of the DEGs and differentially expressed IRGs, we constructed a TF-immune gene regulatory network. We divided CRC patients into two groups according to the results of multivariate Cox regression analysis, constructed IRGPI, then identified multiple clinically independent predictors and risk immune genes in CRC patients. Finally, we evaluated the relationship between immune cell infiltration and IRGPI.

### Identification of IRGs and survival-associated IRGs

**Figure 1:**
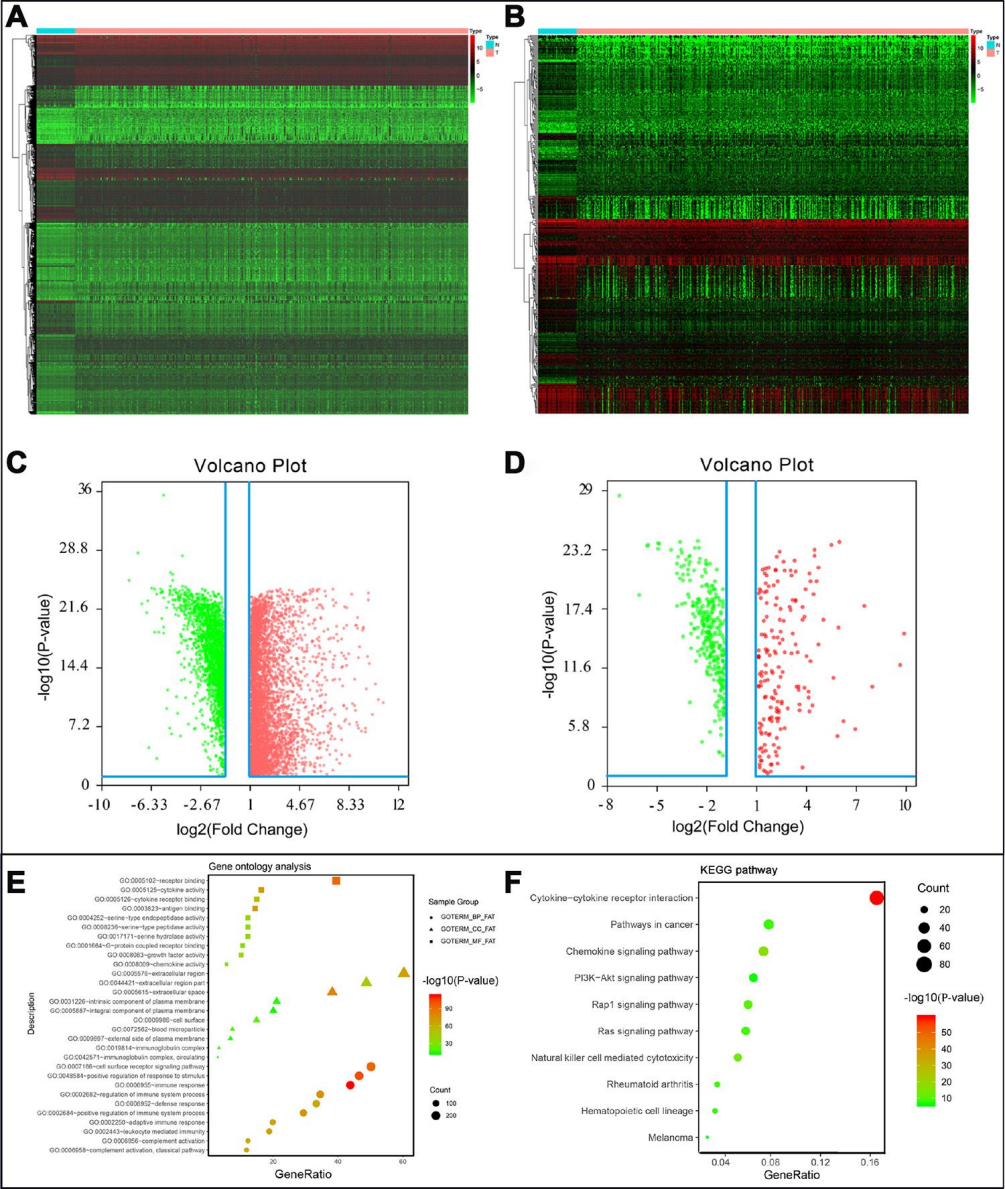
(**A**) Differentially expressed genes, with red representing high expression and green representing low expression. (**B**) Differentially expressed immune-related genes, with red representing high expression and green representing low expression. (**C**) Volcano plot of 6524 differentially expressed genes, with red representing up-regulated and green representing down-regulated. (**D**) Volcano plot of 484 differentially expressed immune-related genes, with red representing up-regulated and green representing down-regulated. (**E**) Gene ontology analysis of differentially immune-related genes, circle presentations biological process, triangle presentations cellular component, square presentations molecular function. (**F**) KEGG pathway analysis of differentially immune-related genes.

**Figure 2:**
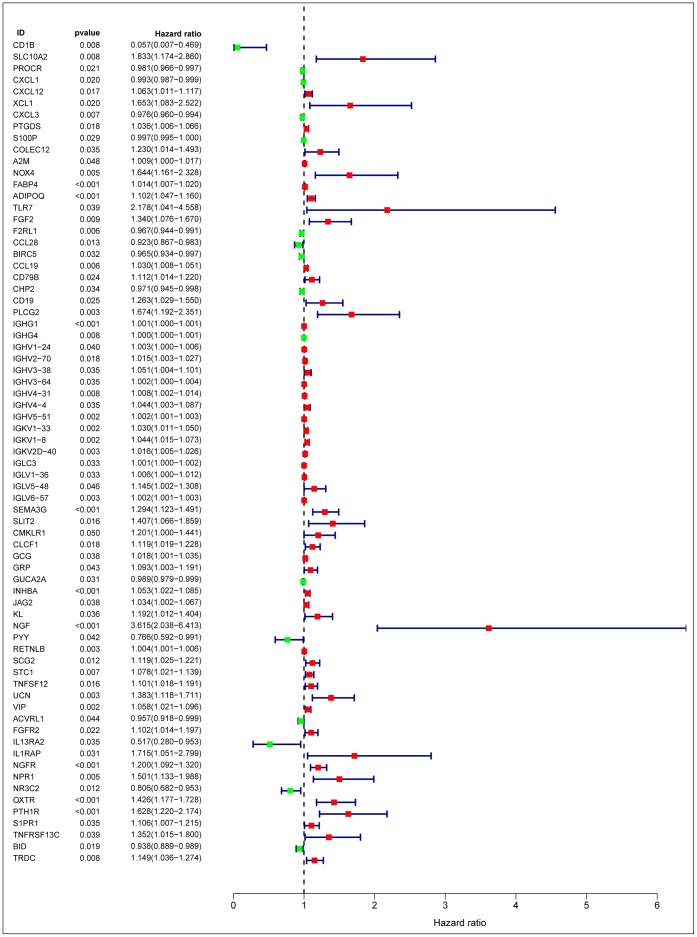
**Forest plot of hazard ratios of prognostically relevant immune genes, revealing prognostic value in CRC.**

**Figure 3:**
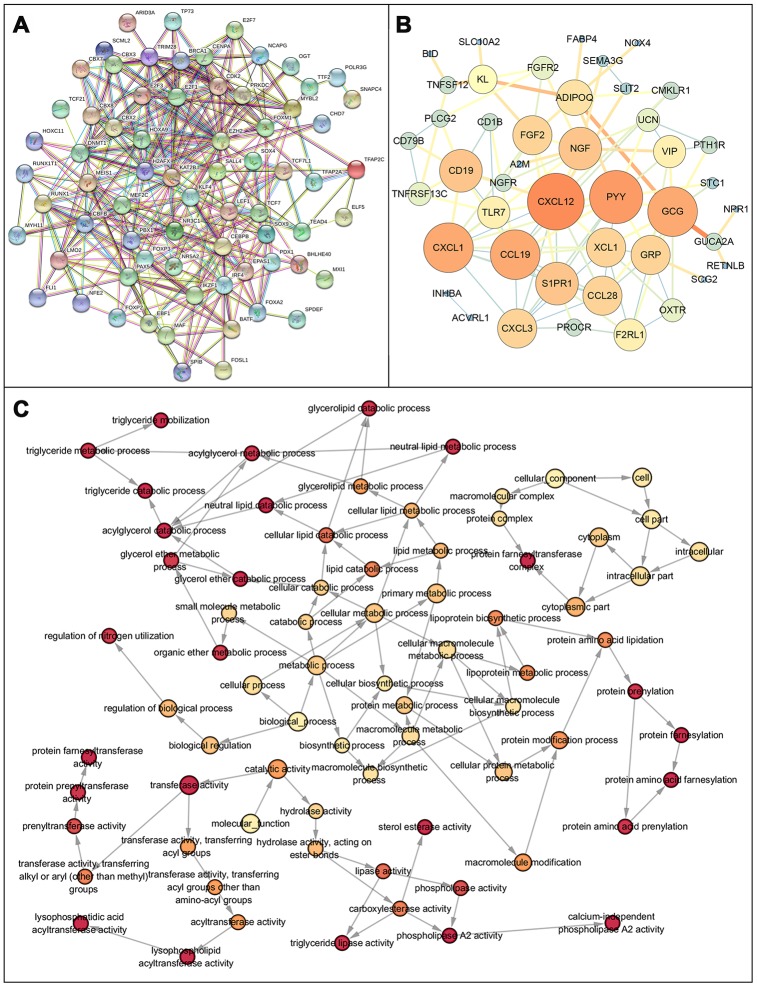
**Protein protein interaction network and GO network of prognosis-related immune genes.** (**A**) Protein-protein interaction network of prognosis-related immune genes, revealing their intrinsic connections. (**B**) The constructed PPIs in Cytoscape, with the size of the nodes showing the degree of connectivity of the immune genes, reveal the hub genes in the network. (**C**) Gene ontology network of prognosis-related immune genes. The color shade of the node represents the p-value, darker colors indicate smaller P values. P < 0.05 indicates statistically significant difference.

**Figure 4:**
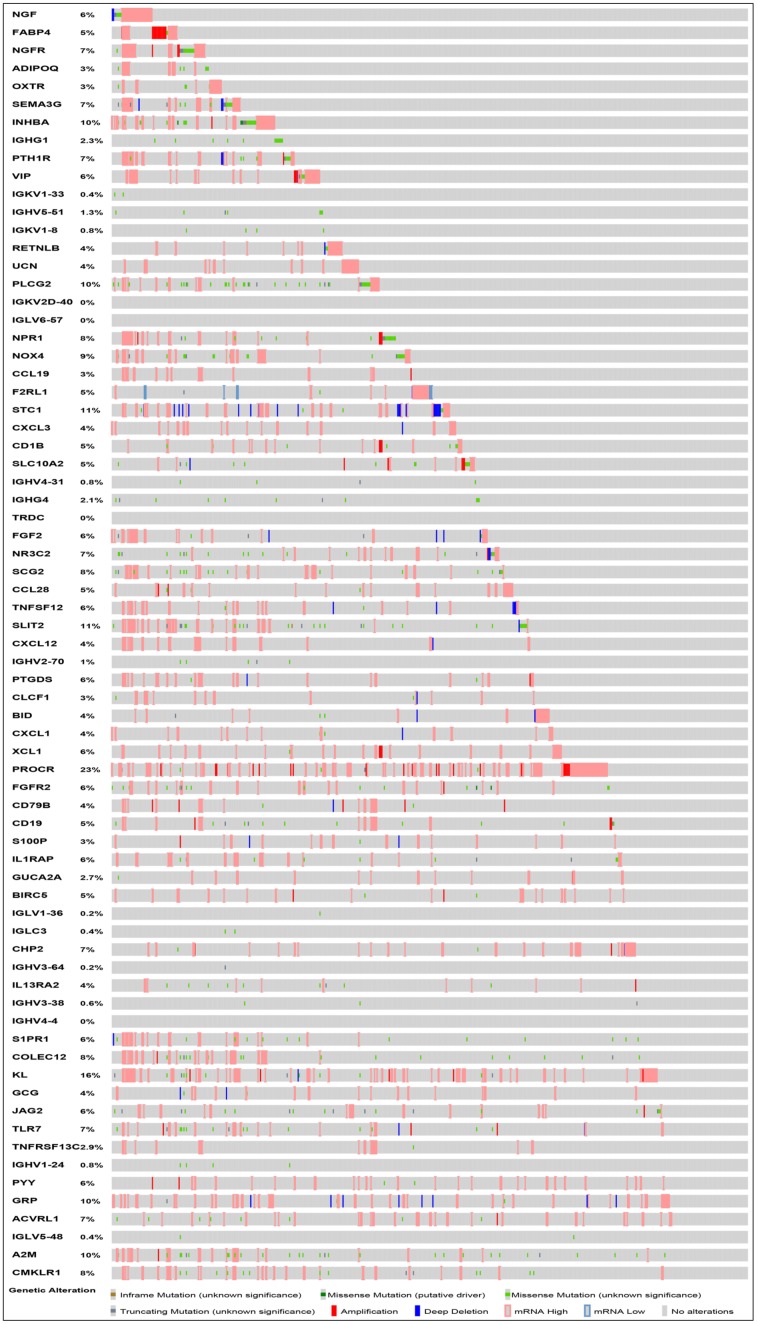
**Mutation landscape of prognosis-related IRGs. PROCR is the gene with the highest mutation frequency. And there were 37 genes with a mutation rate ≥ 5%.**

**Figure 5:**
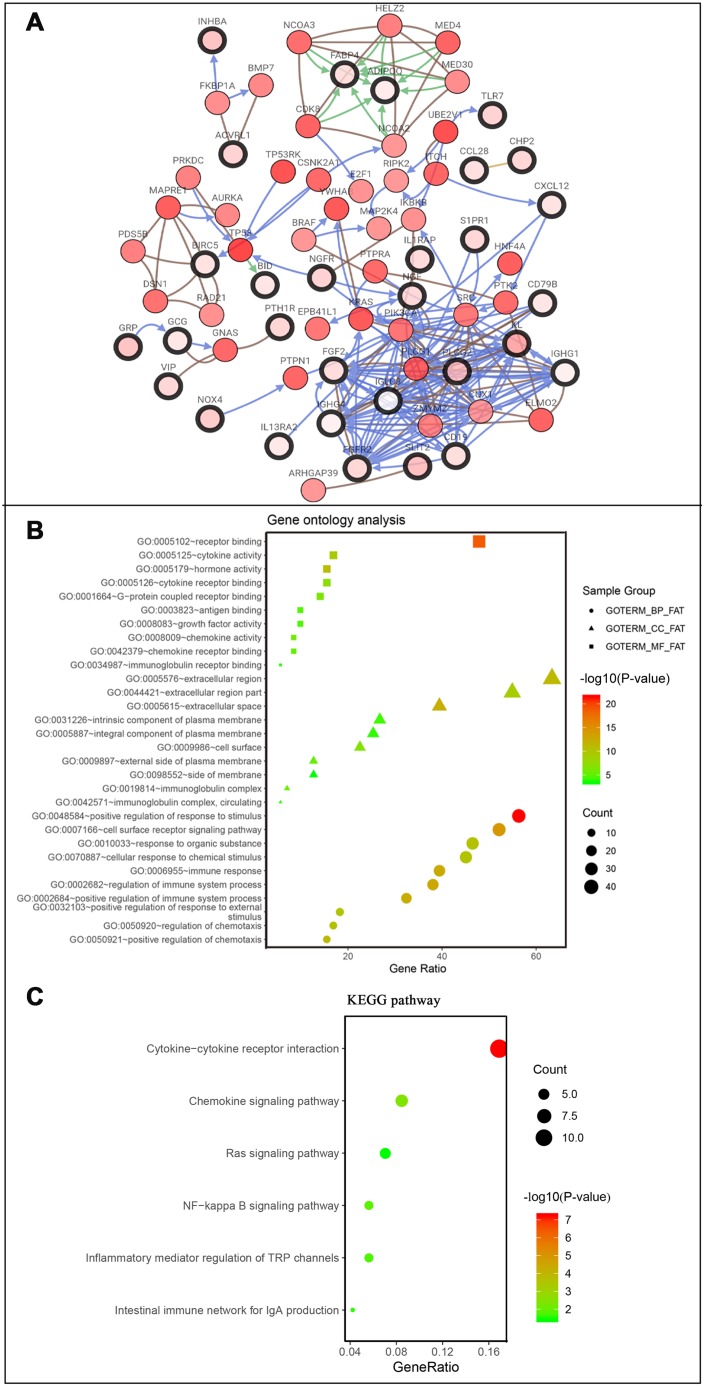
**Co-expression network and gene enrichment analysis of prognostically relevant IRGs.** (**A**) Network of prognostic IRGs and their co-expressed genes, with black-boxed nodes indicating prognostic IRGs and the remaining nodes indicating genes co-expressed with prognostic IRGs. (**B**) Gene ontology analysis and (**C**) KEGG pathway analysis of prognostic IRGs.

After normalization and analysis with the limma package [13] in R software (V3.6.1 https://www.r-project.org), a total of 6524 DEGs were identified (Figure 1A), with the list of immune genes indicating 484 differentially expressed IRGs (Figure 1B). Among these DEGs, 4501 were upregulated while 2023 were downregulated (Figure 1C). Among these differentially expressed IRGs, 173 and 311 were upregulated and downregulated, respectively (Figure 1D). As expected, functional enrichment analysis of these IRGs showed that the most relevant pathways were related to immune, cancer and inflammatory responses. “Immune response,” “extracellular space,” and “receptor binding” were the most frequent biological terms among biological processes, cellular components, and molecular functions, respectively (Figure 1E). The KEGG pathway analysis identified the cytokine-cytokine receptor interaction as the most enriched pathway (Figure 1F). To extract IRGs involved in CRC progression, we chose differentially expressed IRGs that were significantly correlated with clinical outcomes (P<0.05), with a total of 71 survival-related IRGs selected as hub genes (Table 1). Forest plot of hazard ratios for prognostically relevant immune genes indicating the prognostic value of these immune genes in CRC patients is shown in Figure 2. The results of the forest plot indicated that most of the immunity factors were high-risk genes for cancer. A protein-protein interaction (PPI) analysis performed on the immune genes with prognostic value (Figure 3A, 3B), generated a network that revealed that genes, such as C-X-C Motif Chemokine Ligand 12 (CXCL12), Peptide YY (PYY), and Nerve Growth Factor (NGF) were the most highly connected among all genes. A gene ontology (GO) network of these genes was also analyzed using plugins in Cytoscape (Figure 3C). Analysis of mutations performed in the cBioportal database, revealed that many immune genes had inframe, missense, and truncating mutations (Figure 4). In addition, a co-expression network of these genes was constructed (Figure 5A). Functional enrichment analysis of these hub immune genes revealed that biological process was most enriched in positive regulation of response to stimulus, which was consistent with the enrichment results of all differentially expressed immune genes. With regards to cellular component, the extracellular space was the most enriched while receptor binding was the most enriched for molecular function (Figure 5B, 5C).

**Table 1:** General characteristics of CRC-specific immune-related genes.

**Gene symbol**	**logFC**	**FDR**	**HR**	**P-value**
NGF	-1.07762	1.11E-07	3.614874	1.12E-05
FABP4	-1.69607	2.65E-17	1.013513	4.75E-05
NGFR	-1.7335	2.53E-16	1.200272	0.000161
ADIPOQ	-1.99025	9.61E-18	1.101961	0.000222
OXTR	2.970949	1.70E-20	1.426463	0.000285
SEMA3G	-1.98005	7.35E-19	1.294111	0.000355
INHBA	5.410219	6.03E-22	1.052935	0.000678
IGHG1	2.242033	0.000217	1.000558	0.000825
PTH1R	-1.97323	3.73E-19	1.628333	0.000936
VIP	-3.35624	2.99E-21	1.057744	0.001913
IGKV1-33	-2.0014	3.50E-15	1.030337	0.001979
IGHV5-51	-2.04671	3.90E-15	1.001624	0.002062
IGKV1-8	-1.96614	2.74E-15	1.044039	0.002316
RETNLB	-1.11809	4.48E-13	1.003827	0.002823
UCN	2.370658	2.94E-17	1.383005	0.002826
PLCG2	-1.81429	6.39E-19	1.67407	0.002941
IGKV2D-40	-1.04607	1.15E-10	1.015759	0.003079
IGLV6-57	-1.60553	4.76E-12	1.001968	0.003346
NPR1	-1.70244	3.59E-16	1.50082	0.00463
NOX4	3.263304	5.66E-18	1.643741	0.00513
CCL19	-2.15933	9.35E-15	1.029576	0.005852
F2RL1	-1.11351	1.65E-17	0.966769	0.006461
STC1	2.269389	2.56E-16	1.078088	0.0072
CXCL3	3.047496	4.68E-18	0.976418	0.007315
CD1B	1.090892	0.012274	0.057461	0.007658
SLC10A2	-7.37303	2.19E-25	1.832561	0.007661
IGHV4-31	-1.69851	9.17E-11	1.008218	0.007668
IGHG4	1.769556	0.02942	1.000463	0.008256
TRDC	-1.85546	1.40E-15	1.14881	0.008409
FGF2	-1.37298	1.56E-11	1.340343	0.009
NR3C2	-2.59095	7.31E-22	0.8063	0.011706
SCG2	-2.14034	2.39E-19	1.118764	0.012272
CCL28	-1.9804	6.46E-18	0.923295	0.012702
TNFSF12	-1.14925	1.37E-12	1.101101	0.015819
SLIT2	-1.69869	2.77E-14	1.407394	0.016025
CXCL12	-2.54566	4.83E-21	1.062678	0.01692
IGHV2-70	-1.45759	9.83E-11	1.014614	0.017613
PTGDS	-1.35298	7.22E-12	1.035543	0.01783
CLCF1	1.016493	3.25E-10	1.118713	0.018123
BID	1.147326	2.83E-18	0.937667	0.018657
CXCL1	2.94518	3.55E-17	0.993112	0.019735
XCL1	-1.02275	1.65E-09	1.652723	0.019766
PROCR	1.21046	4.01E-11	0.981309	0.020768
FGFR2	-2.15905	2.28E-20	1.101962	0.021634
CD79B	-2.05831	1.30E-16	1.112374	0.023507
CD19	-1.8624	2.94E-11	1.263127	0.025436
S100P	2.517715	3.23E-16	0.997402	0.028846
IL1RAP	1.053287	5.06E-13	1.71503	0.030956
GUCA2A	-5.04559	7.70E-22	0.988757	0.031143
BIRC5	1.625605	2.98E-19	0.96511	0.031986
IGLV1-36	-1.61723	5.61E-15	1.006035	0.033026
IGLC3	-1.8978	5.95E-13	1.000929	0.033267
CHP2	-3.81304	1.80E-21	0.971156	0.033524
IGHV3-64	-1.38936	7.61E-14	1.00211	0.034568
IL13RA2	1.365327	0.000112	0.516776	0.034577
IGHV3-38	-1.95173	1.53E-14	1.050918	0.03483
IGHV4-4	-2.60452	1.40E-15	1.043988	0.035024
S1PR1	-1.03942	2.45E-13	1.105949	0.035328
COLEC12	-1.7569	5.08E-18	1.230376	0.035387
KL	-1.1424	5.26E-16	1.191899	0.035919
GCG	-4.9816	5.83E-22	1.017926	0.03803
JAG2	2.065779	1.29E-17	1.033744	0.038079
TLR7	-1.74294	1.68E-17	2.178313	0.038773
TNFRSF13C	-1.60074	1.90E-08	1.35185	0.03901
IGHV1-24	-1.00006	3.78E-08	1.003218	0.040381
PYY	-5.71	6.50E-22	0.766028	0.042196
GRP	2.434638	9.19E-09	1.093047	0.042615
ACVRL1	-1.50426	2.54E-18	0.957411	0.044483
IGLV5-48	-2.84863	1.67E-15	1.144904	0.046039
A2M	-1.26394	7.65E-17	1.008646	0.047604
CMKLR1	-1.43947	1.61E-16	1.200541	0.049798

FC: fold change; FDR: false discovery rate; HR: hazard ratio.

### Gene set enrichment analysis (GSEA)

**Figure 6:**
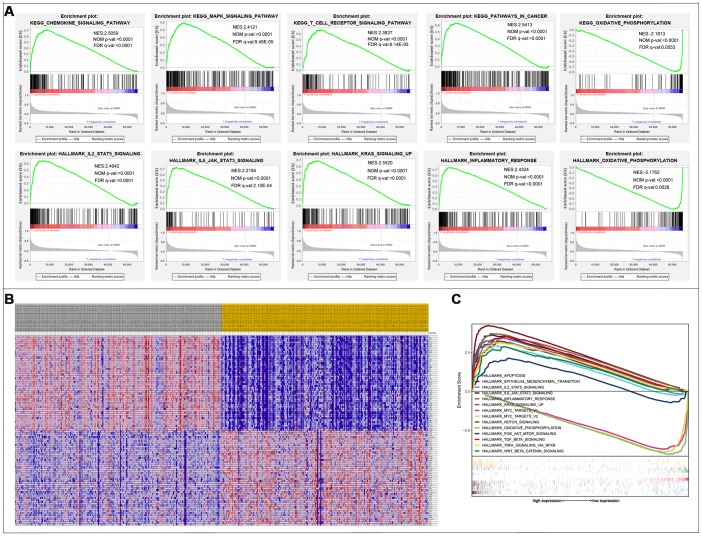
**Gene Set Enrichment Analysis.** (**A**) 10 significantly enriched pathways. (**B**) Cluster heatmap of top 50 high and low expressed genes in all samples. (**C**) Comparative analysis of the top 10 significantly enriched pathways.

The results of the GSEA enrichment analysis on the selected differential genes were relatively similar to those from Kyoto encyclopedia of genes and genomes (KEGG) enrichment analysis of differentially immune genes in the DAVID database. A significant number of genes were enriched in pathways related to immunity, cancer, among others. KEGG results showed that a majority of the genes were enriched in PATHWAYS-IN-CANCER and T-CELL-RECEPTOR-SIGNALING-PATHWAY, while the Hallmark background showed enrichment mainly for INFLAMMATORY-RESPONSE, EPITHELIAL-MESENCHYMAL-TRANSITION, and KRAS-SIGNALING-UP (Figure 6). Results were screened for a subset of significant differences revealing that the immunity genes were largely linked to CRC through the aforementioned, well-defined pathways (Tables 2, 3). This lays a foundation for the subsequent application of CRC immunotherapy.

**Table 2:** GSEA results showing pathways enriched in the top or bottom of the ranked list (part 1).

**gsea_report_for_h_1569243707744**						
**PATHWAY**	**SIZE**	**ES**	**NES**	**NOM p-val**	**FDR q-val**	**FWER p-val**
KEGG_REGULATION_OF_ACTIN_CYTOSKELETON	213	0.66964203	2.5797722	0	0	0
KEGG_FOCAL_ADHESION	199	0.75337255	2.5684252	0	0	0
KEGG_GAP_JUNCTION	90	0.67211634	2.565771	0	0	0
KEGG_PATHWAYS_IN_CANCER	325	0.6266958	2.541297	0	0	0
KEGG_VASCULAR_SMOOTH_MUSCLE_CONTRACTION	115	0.68861216	2.5375874	0	0	0
KEGG_CHEMOKINE_SIGNALING_PATHWAY	188	0.7085286	2.5059814	0	0	0
KEGG_BASAL_CELL_CARCINOMA	55	0.6802519	2.3073714	0	4.55E-05	0.001
KEGG_RENAL_CELL_CARCINOMA	70	0.65006334	2.3362064	0	4.73E-05	0.001
KEGG_ADHERENS_JUNCTION	73	0.6770658	2.3461874	0	4.91E-05	0.001
KEGG_MELANOGENESIS	101	0.615403	2.3612275	0	5.12E-05	0.001
KEGG_FC_EPSILON_RI_SIGNALING_PATHWAY	79	0.63750756	2.3671906	0	5.34E-05	0.001
KEGG_CELL_ADHESION_MOLECULES_CAMS	131	0.76282525	2.3726993	0	5.58E-05	0.001
KEGG_LEUKOCYTE_TRANSENDOTHELIAL_MIGRATION	116	0.6907626	2.3731625	0	5.85E-05	0.001
KEGG_T_CELL_RECEPTOR_SIGNALING_PATHWAY	108	0.6633691	2.383714	0	6.14E-05	0.001
KEGG_PANCREATIC_CANCER	70	0.63705605	2.2872703	0	6.39E-05	0.002
KEGG_ECM_RECEPTOR_INTERACTION	84	0.8301615	2.3839304	0	6.47E-05	0.001
KEGG_NEUROACTIVE_LIGAND_RECEPTOR_INTERACTION	271	0.62170815	2.2894022	0	6.60E-05	0.002
KEGG_ADIPOCYTOKINE_SIGNALING_PATHWAY	67	0.6279159	2.2899668	0	6.82E-05	0.002
KEGG_JAK_STAT_SIGNALING_PATHWAY	155	0.61718726	2.3874989	0	6.83E-05	0.001
KEGG_HEMATOPOIETIC_CELL_LINEAGE	85	0.7675451	2.2916093	0	7.05E-05	0.002
KEGG_PROSTATE_CANCER	89	0.6342127	2.3921416	0	7.23E-05	0.001
KEGG_HYPERTROPHIC_CARDIOMYOPATHY_HCM	83	0.6716272	2.2937078	0	7.30E-05	0.002
KEGG_DILATED_CARDIOMYOPATHY	90	0.7019853	2.3925047	0	7.68E-05	0.001
KEGG_DORSO_VENTRAL_AXIS_FORMATION	24	0.7876579	2.3928838	0	8.19E-05	0.001
KEGG_AXON_GUIDANCE	129	0.6520283	2.4046438	0	8.78E-05	0.001
KEGG_MAPK_SIGNALING_PATHWAY	267	0.5887808	2.4121554	0	9.45E-05	0.001
KEGG_CYTOKINE_CYTOKINE_RECEPTOR_INTERACTION	264	0.67623246	2.4139163	0	1.02E-04	0.001
KEGG_CALCIUM_SIGNALING_PATHWAY	177	0.63363373	2.4335191	0	1.12E-04	0.001
KEGG_MELANOMA	71	0.6444286	2.4477468	0	1.23E-04	0.001
KEGG_FC_GAMMA_R_MEDIATED_PHAGOCYTOSIS	96	0.64547414	2.2792652	0	1.27E-04	0.003
**gsea_report_for_l_1569243707744**						
**PATHWAY**	**SIZE**	**ES**	**NES**	**NOM p-val**	**FDR q-val**	**FWER p-val**
KEGG_PARKINSONS_DISEASE	128	-0.7638063	-2.1532152	0	0.003386	0.014
KEGG_OXIDATIVE_PHOSPHORYLATION	131	-0.7945146	-2.181362	0	0.00531	0.011
KEGG_RIBOSOME	87	-0.9401583	-2.0572634	0	0.006679	0.041
KEGG_HUNTINGTONS_DISEASE	180	-0.62558216	-2.0771437	0	0.006703	0.032

**Table 3:** GSEA results showing pathways enriched in the top or bottom of the ranked list (part 2).

**gsea_report_for_h_1569251519150**						
**PATHWAY**	**SIZE**	**ES**	**NES**	**NOM p-val**	**FDR q-val**	**FWER p-val**
HALLMARK_KRAS_SIGNALING_UP	200	0.68496144	2.5519905	0	0	0
HALLMARK_MYOGENESIS	200	0.7163382	2.4803855	0	0	0
HALLMARK_INFLAMMATORY_RESPONSE	200	0.74277735	2.4324496	0	0	0
HALLMARK_IL2_STAT5_SIGNALING	200	0.64027786	2.404173	0	0	0
HALLMARK_APICAL_JUNCTION	200	0.66379976	2.2760096	0	0	0
HALLMARK_COMPLEMENT	200	0.64504373	2.269838	0	0	0
HALLMARK_EPITHELIAL_MESENCHYMAL_TRANSITION	198	0.84459037	2.4160647	0	0	0
HALLMARK_UV_RESPONSE_DN	142	0.7505294	2.5657384	0	0	0
HALLMARK_COAGULATION	138	0.6745744	2.3511252	0	0	0
HALLMARK_TGF_BETA_SIGNALING	54	0.7044607	2.3415003	0	0	0
HALLMARK_ANGIOGENESIS	36	0.78319705	2.304125	0	0	0
HALLMARK_IL6_JAK_STAT3_SIGNALING	87	0.7255876	2.219405	0	2.10E-04	0.003
HALLMARK_HEDGEHOG_SIGNALING	36	0.70793414	2.135469	0	8.37E-04	0.008
HALLMARK_ALLOGRAFT_REJECTION	200	0.6927329	2.1372313	0.0020243	9.02E-04	0.008
HALLMARK_NOTCH_SIGNALING	32	0.64948887	2.1142936	0	0.001424	0.016
HALLMARK_APOPTOSIS	161	0.5897472	2.1039493	0	0.0015353	0.018
HALLMARK_HYPOXIA	197	0.5773829	2.0876963	0	0.0018728	0.024
HALLMARK_APICAL_SURFACE	44	0.6299601	2.0555112	0.0019011	0.0022883	0.031
**gsea_report_for_l_1569251519150**						
**PATHWAY**	**SIZE**	**ES**	**NES**	**NOM p-val**	**FDR q-val**	**FWER p-val**
HALLMARK_OXIDATIVE_PHOSPHORYLATION	200	-0.81177884	-2.1761758	0	0.0027603	0.007
HALLMARK_MYC_TARGETS_V1	199	-0.7470366	-1.9788883	0.004065	0.0174095	0.064
HALLMARK_MYC_TARGETS_V2	58	-0.79232734	-1.9486467	0.0041068	0.0176199	0.085

### Identification of transcription factors and immune gene regulatory networks

**Figure 7:**
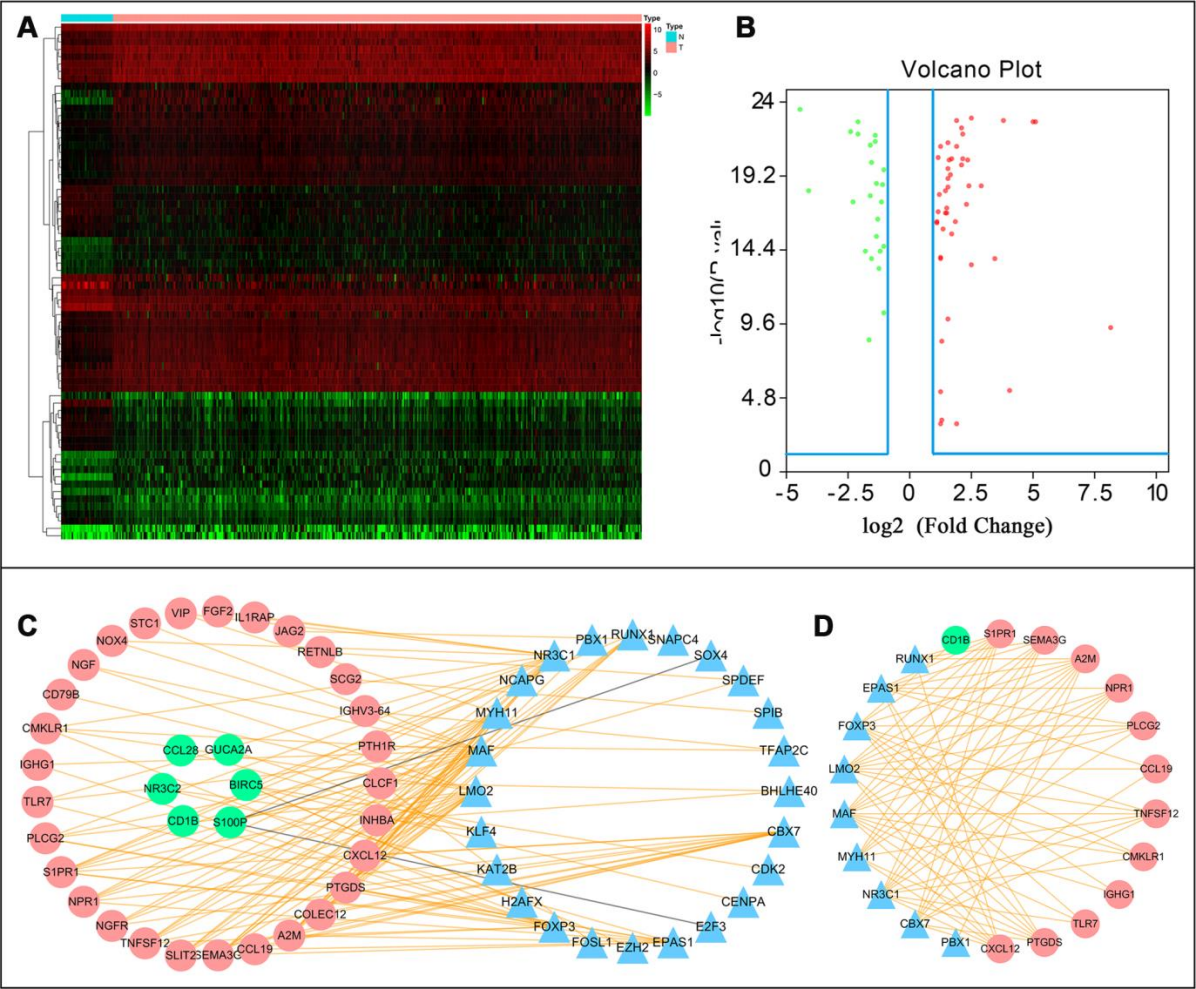
**Transcription factor mediated regulatory network.** Differentially expressed transcription factors (TFs) (**A**) hetmap and (**B**) Volcano plot. (**C**) Regulatory network constructed based on clinically relevant TFs and IRGs. (**D**) Most significant modules in regulatory networks.

A total of 70 differentially expressed transcription factors were selected from the tumor-associated transcription factors downloaded in the Cistrome Cancer Database. Among them, 46 and 24 were upregulated and downregulated, respectively (Figure 7A and 7B). Subsequently, we developed a transcription factor-immune gene regulatory network and generated a map (Figure 7C), which showed a strong correlation module based on MCODE plugin (Figure 7D). The results revealed 3 high-risk immune genes, namely Prostaglandin D2 Synthase (PTGDS), Alpha-2-Macroglobulin (A2M), and Sphingosine-1-Phosphate Receptor 1 (S1PR1), as well as 2 TFs, Forkhead Box P3 (FOXP3) and Endothelial PAS Domain Protein 1(EPAS1), with strong association to prognosis of CRC. Literature search showed that A2M expression is downregulated in tumors compared to normal adjacent samples [14], which was consistent with our findings. We, therefore, hypothesized that A2M and other immune genes are inhibited by transcription factors, resulting in decreased expression, and rendering the low expression of immune genes a high-risk factor in tumor development. This also illustrates, to some extent, the accuracy of our constructed network. However, further analysis is required to explore the implication of these immune genes and the corresponding transcription factors in CRC to guide designing and development of immunotherapy of CRC.

### Construction of a clinical prognostic model

**Figure 8:**
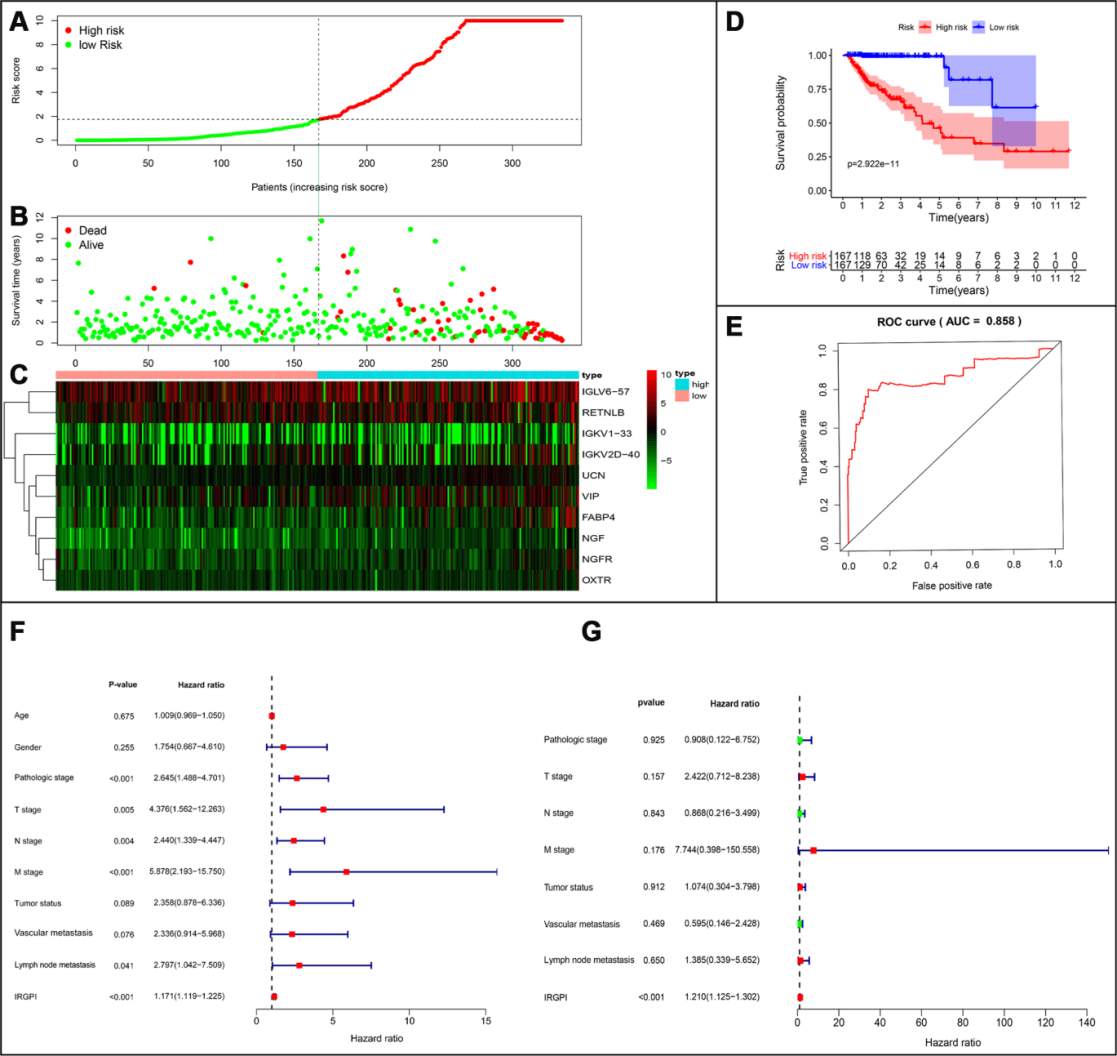
**Establishment of prognostic index based on prognostic related immune genes.** (**A**) Rank of prognostic index and distribution of groups. (**B**) Survival status of patients in different groups. (**C**) Heatmap of expression profiles of included genes. (**D**) Five-year survival was significantly lower in the high-risk group. (**E**) Survival-dependent receiver operating characteristic (ROC) curve validation of prognostic value of the prognostic index. (**F**) Univariate regression and (**G**) multiple regression analysis of colorectal cancer.

Based on the results of multivariate Cox regression analysis, we developed a prognostic index to group CRC patients into two: high and low risk, and constructed a risk curve (Figure 8A–8C). A higher risk score indicated a shorter survival time for patients. This immune-based prognostic index could be an important tool for distinguishing among CRC patients based on potential discrete clinical outcomes (Figure 8D). The following formula was used: [Expression level of FABP4 * (0.0169)] + [Expression level of IGKV1-33 * (0.0288)] + [Expression level of IGKV2D-40 * (0.01050)] + [Expression level of IGLV6-57 * (0.0021)] + [Expression level of NGF * 1.1134] + [Expression level of RETNLB * (0.0045)] + [Expression level of UCN * (0.3985)] + [Expression level of VIP * (0.0617)] + [Expression level of NGFR * (-0.2621)] + [Expression level of OXTR * (0.2656)].Thus, we show that this prognostic index can effectively and accurately stratify CRC patients. The area under the curve (AUC) of the receiver operating characteristic (ROC) was 0.858 (Figure 8E), indicating a high prognostic performance of the IRGs in survival surveillance. Results from Cox regression analysis of univariate and multivariate factors are outlined in Figure 8F, 8G. For univariate risk analysis, lymph node and, vascular metastases, tumor status, CRC pathological stage, TNM stage and IRGPI were found to be independent predictors. However, after computational analysis of all related clinical factors, we found that IRGPI were an independent predictor.

### Clinical correlation analysis

**Figure 9:**
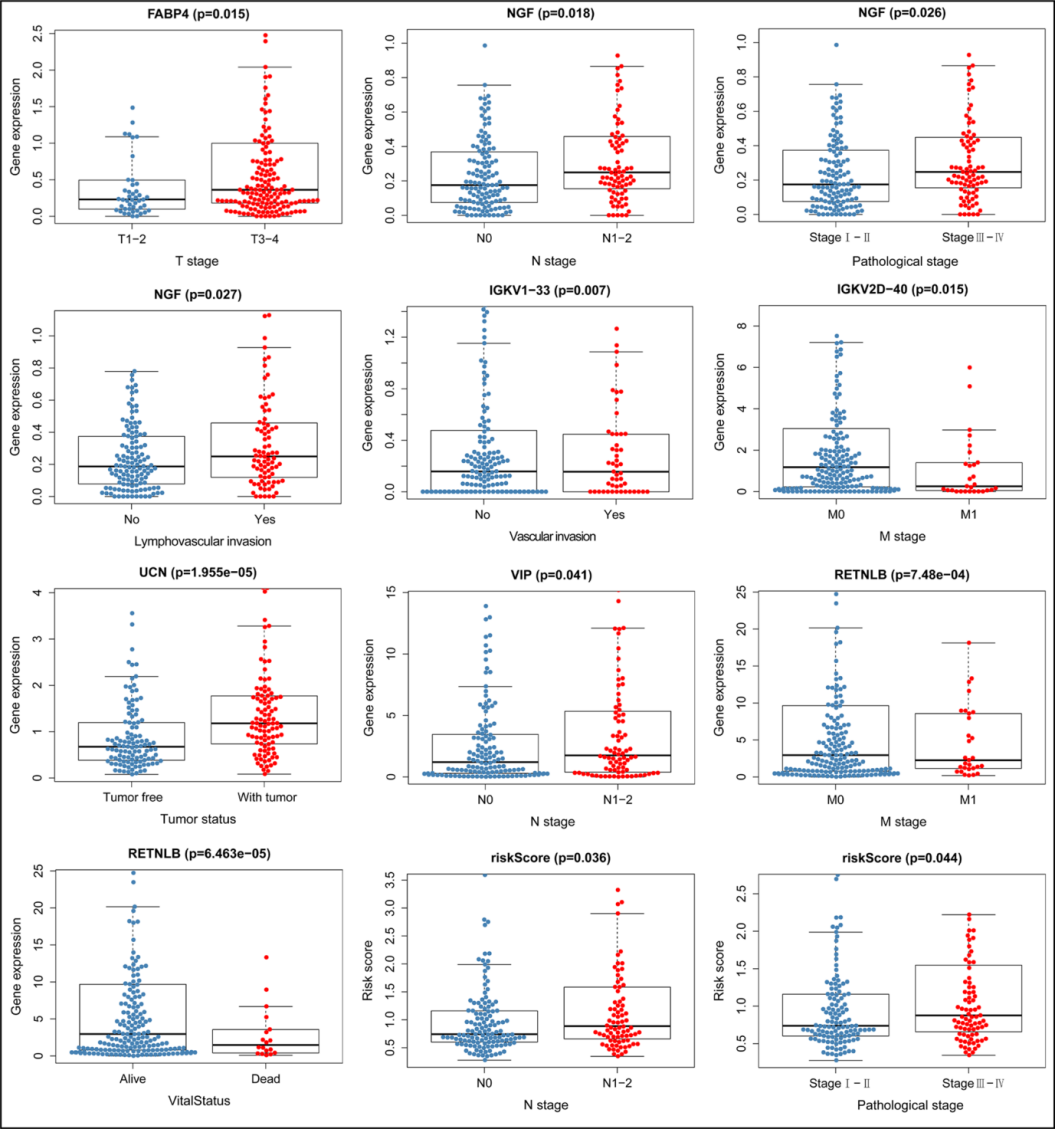
**Relationship between immune gene expression and clinicopathological factors in CRC (P < 0.05).**

**Figure 10:**
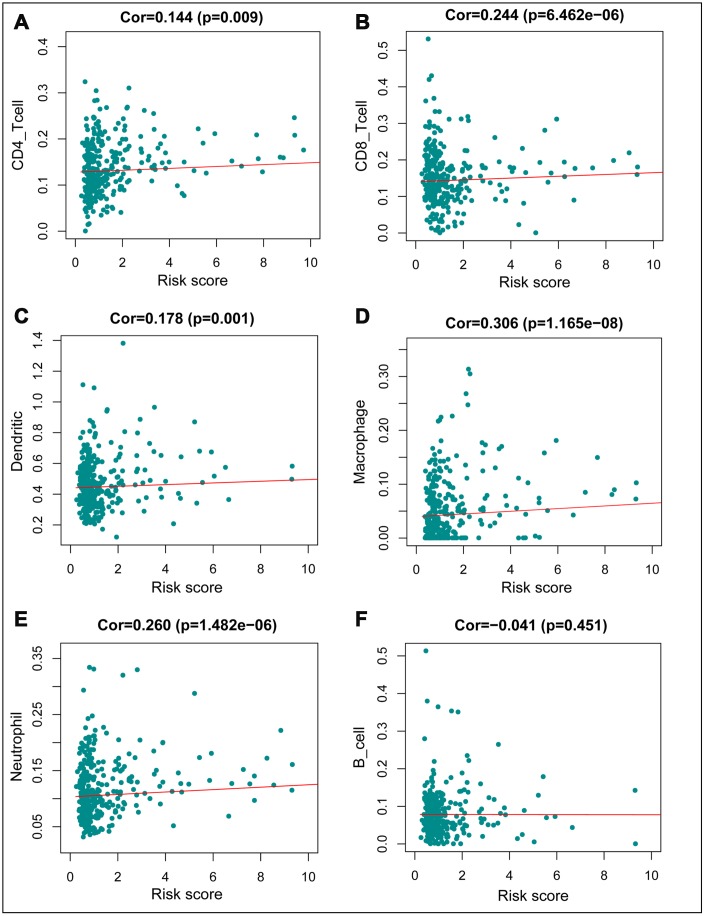
**Relationships between the immune–related prognostic index and infiltration abundances of six types of immune cells.** (**A**) CD4 T cells; (**B**) CD8 T cells; (**C**) dendritic cells; (**D**) macrophages; (**E**) neutrophils; and (**F**) B cells. The correlation was performed by using Pearson correlation analysis. P < 0.05 was considered statistically significant.

We constructed models to analyze the relationship between immunity genes with clinical and demographic characteristics, including age, sex, pathological tumor stage, TNM stage of the International Union Against Cancer, lymphatic, vascular invasion, and tumor burden. A summary of results from the computational analysis is shown in Table 4, while genes with significant statistical differences are shown in Figure 9. Thereafter, we assessed the association between individual gene expression and the corresponding clinical traits. For example, NGF was correlated with lymphatic metastasis, tumor stage, and N stage. We also assessed the relationship between immune cell infiltration and the IRGPI, to determine whether immune genes accurately reflected the state of tumor immune environment. We found a positive correlation between IRGPI with CD4+T, CD8+T, and dendritic cells, as well as macrophages and neutrophils, while this factor was negatively correlated with B cells, although no significant differences (P > 0.05) were observed (Figure 10).

**Table 4:** Relationships between the expressions of the immune-related genes and the clinicopathological factors incolorectal cancer.

**Gene symbol**	**Age (≥60/<60)**	**Gender (male/female)**	**T stage (T3–T4/ T1–T2)**	**N stage (N1–3/ N0)**	**M stage (M1/ M0)**	**Pathological stage (IV-III/ I–II)**	**VitalStatus (dead/alive)**	**Tumor status (with tumor/tumor free)**	**Vascular invasion (yes/no)**	**Lymphovascular invasion (yes/no)**
	**t(P)**	**t(P)**	**t(P)**	**t(P)**	**t(P)**	**t(P)**	**t(P)**	**t(P)**	**t(P)**	**t(P)**
FABP4	1.715 (0.089)	0.684 (0.495)	**-2.456** **(0.015)**	-0.662 (0.508)	-1.099 (0.280)	-0.562 (0.575)	-1.2 (0.244)	0.842 (0.401)	-1.256 (0.213)	-0.43 (0.668)
IGKV1-33	0.525 (0.600)	-0.995 (0.321)	0.606 (0.546)	0.557 (0.578)	1.319 (0.195)	0.676 (0.500)	1.638 (0.112)	0.558 (0.578)	**2.747** **(0.007)**	0.79 (0.430)
IGKV2D-40	1.676 (0.097)	1.433 (0.155)	-1.19 (0.236)	-0.812 (0.419)	**2.456** **(0.015)**	-0.74 (0.461)	-0.683 (0.502)	0.384 (0.702)	-0.541 (0.591)	-0.348 (0.729)
IGLV6-57	0.951 (0.343)	0.218 (0.828)	0.637 (0.526)	0.921 (0.358)	0.939 (0.354)	1.135 (0.258)	0.194 (0.848)	-1.879 (0.062)	0.211 (0.834)	-0.408 (0.684)
NGF	1.348 (0.180)	0.726 (0.469)	-1.472 (0.145)	**-2.385** **(0.018)**	-1.581 (0.124)	**-2.251** **(0.026)**	-1.329 (0.201)	0.935 (0.351)	-1.675 (0.099)	**-2.232** **(0.027)**
RETNLB	0.899 (0.370)	0.36 (0.719)	1.244 (0.219)	1.554 (0.122)	**3.423** **(<0.001)**	1.611 (0.109)	**4.077** **(<0.001)**	0.964 (0.336)	0.944 (0.347)	1.073 (0.285)
UCN	-1.101 (0.272)	-0.948 (0.344)	-0.864 (0.390)	-1.498 (0.136)	-0.448 (0.657)	-1.633 (0.105)	-1.443 (0.166)	**-4.387** **(<0.001)**	-0.308 (0.759)	-0.616 (0.539)
VIP	0.437 (0.663)	1.431 (0.154)	-1.748 (0.084)	**-2.059** **(0.041)**	-0.401 (0.691)	-1.769 (0.079)	0.169 (0.867)	1.723 (0.087)	-1.334 (0.187)	-1.677 (0.095)
NGFR	1.28 (0.203)	1.838 (0.068)	-1.591 (0.115)	-1.562 (0.121)	-0.625 (0.535)	-1.453 (0.148)	-0.834 (0.412)	1.892 (0.060)	-1.087 (0.281)	-1.325 (0.187)
OXTR	0.789 (0.431)	-1.223 (0.223)	-1.055 (0.294)	-1.258 (0.211)	0.36 (0.720)	-1.217 (0.226)	-0.487 (0.630)	1.587 (0.114)	0.699 (0.486)	-0.374 (0.709)
riskScore	1.523 (0.130)	0.976 (0.331)	-1.501 (0.136)	**-2.124** **(0.036)**	-0.764 (0.450)	**-2.035** **(0.044)**	-1.581 (0.130)	-0.402 (0.688)	-1.422 (0.161)	-1.659 (0.100)

Note: t: t value of student's t test; P: P-value of student's t test.

## DISCUSSION

The role of IRGs in cancer development, especially CRC, has not been fully studied. In this study, we performed a comprehensive integrated analyzed of IRGs involved in CRC and interpreted their clinical value. Our findings reveal the underlying molecular features and unearth the potential impact of IRGs in immunotherapy against CRC. We used a large number of clinical and expression profiling samples, identified and normalized multiple clinical traits, analyzed transcription factor information, and performed a induction of angiogenesis, resistance to cell death, evasion of growth suppressors, and proliferative signals, and immortalization of replication, acquired during tumor formation [16]. Studies have demonstrated that tumor cells also exhibit functions such as immune evasion, while environmental and genetic factors have also been implicated in tumor progression. Gene mutations as well as the recently-discovered large number of micro and long non-coding RNAs, also play a large role in tumor development [17, 18]. Currently, CRC is mainly treated using surgery, radiation, and chemotherapy. However, these approaches are not effective for advanced and metastatic tumors, with nearly 50% of the patients subjected to these treatments succumbing to CRC due to recurrence or metastasis. This has necessitated further research aimed at developing novel and effective immunotherapies for CRC [19]. Immunotherapy has become the primary treatment modality for many types of solid cancers, with various types of this therapy reported to successfully manage the condition, especially in patients with metastatic cancer. Such therapies produce long-lasting control against tumors, and are therefore considered superior to other approaches [20]. Recently, nivolumab and pembrolizumab, drugs that target PD1, have been applied for management of metastatic CRC. Immunotherapeutic approaches that can control the development of CRC possess the potential to provide long-term durable remission of CRC patients [5, 21].

The origin of cancer cells, and progression of the disease, depends on the interaction between genetic, epigenetic and epitranscriptomic alterations. CRC is a tumor with a strong epigenetic component, which is particularly important in promoting CRC progression [22]. Mutations in tumor cell genes have been found to activate cytotoxic lymphocytes, thereby inhibiting tumor growth. Conversely, persistent inflammatory responses caused by deregulated immune function, toxins from environment and infections promote tumor cell growth, their survival, invasion, and dissemination [23]. Most human tumors are associated with infiltration of different types of immune cells. In addition, the interaction between host immune cells with other components of the tumor microenvironment is a key determinant of various tumor processes. Therefore, regulating this interaction could provide an opportunity combined analysis of immune cell infiltration, to generate a concrete conclusion. We identified several IRGs that were significantly associated with CRC progression and are therefore capable of being cancer biomarkers. We used bioinformatics tools to explore the regulatory network and specific molecular mechanisms in greater depth, and assessed immune cell infiltration as well as disease outcomes based on individualized immune prognostic indicators constructed from the analyzed differential IRGs.

Zhang et al reported differences in immune cell infiltration and immunophenotype between right- and left-side CRC, and how these influence prognosis of CRC patients [15]. Conversely, our study focused on constructing a very practical immunolabeling tool for immune-related genes to stratify CRC patients and predict prognosis, and can therefore be used to reflect the level of immune cell infiltration. The IRGPI presented in this study is simple to calculate and may be an ideal tool for implementation in daily clinical practice. In fact, clinicians can apply this tool during monitoring of prognosis in CRC patients in a simpler way. At the same time, the information herein, including immune cell infiltration of patients, can be combined and used to further improve immunotherapy strategies and greatly promote development of new therapies for CRC patients.

Recent developments in medical technology have promoted research into the key drivers of cancer and revealed effective approaches to fight the disease. Infiltration of the tumor environment with many inflammatory cells often leads to cancer development. The hallmarks of cancer comprise six biological processes, activation of invasion and metastasis, for design and development of effective therapeutic and preventive interventions [24]. This study, therefore, explored the implication of various immune features on CRC clinical outcomes. Particularly, gene and functional analyses show that many immune cells may be involved in CRC, and most of these genes are enriched in the chemokine signaling and cytokine-cytokine receptor interaction pathways.

Chemokines are key players in the innate immune system of the human body. They primarily control migration and localization of immune cells to sites of infection and inflammation and also initiate and control adaptive immune responses thereby regulating cancer development and metastasis [25, 26]. Chemokines, and their receptors, are associated with CRC metastasis. In fact, expression of their receptors including C-X-C Motif Chemokine Receptor 4 (CXCR4) and C-C Motif Chemokine Receptor 6 (CCR6), has been proposed as a potential predictor of CRC recurrence, poor survival, and liver metastasis [27, 28]. These factors can, therefore, be exploited to enable monitor the survival and metastasis of CRC patients. Our bioinformatics analyses revealed that alterations in the immunogenome can contribute to initiation of CRC through several inflammatory pathways.

To better understand the relationship between IRGs and prognosis, we analyzed overall survival time as the clinical endpoint. Many pathways involved in IRGs have been implicated in immunity and cancer. The most highly correlated pathways with survival-associated IRGs were those related to PATHWAYS-IN-CANCER, MAPK, and KRAS. In fact, we observed that most of the prognosis-related immune genes were associated with tumor-related pathways. The mitogen-activated protein kinase (MAPK) pathway regulates multiple cellular processes including migration, differentiation, and proliferation. Several subfamilies of this pathway regulates gene mutations, growth factor-related apoptosis and induction of apoptosis in CRC by activating the Ras/Raf/MEK/ERK pathway. Previous studies have shown that the MAPK pathway modulates metastasis and invasion of CRC [29]. KRAS belongs to the Kirsten ras oncogene homolog of the RAS gene family, and was one of the first genes to be mutated in a variety of cancers. The KRAS encoded transforming protein has been associated with various malignancies, including lung adenocarcinoma, pancreatic ductal carcinoma, and CRC. And the specific site of mutation varies among cancers [30]. Understanding how these mutations arise could provide insights into cancer development, which is a prerequisite for designing effective preventive cancer strategies. In our study, we found downregulation of many genes following KRAS activation. This was consistent with our expectations, and is expected to promote further investigations into the specific relationship among immune genes with KRAS for better CRC diagnosis and treatment [31]. Important information concerning expression profiles, prognostic value and mutational status of these IRGs of prognostic value was also obtained and this will enhance further studies.

The roles played by IRGs in clinical prognosis of CRC were further explored, based on the constructed TF-mediated network. According to this analysis, A2M, CXCL12, S1PR1, among others were identified as the key TFs in the network. This TF-IRG regulatory network is expected to guide future mechanistic analyses in the field. Results from previous studies corroborate the findings herein, thereby validating their reliability. CXCL12, a ligand for CXCR4, encodes a protein that acts as a G protein-coupled receptor and is involved in cell processes including immune surveillance, inflammatory responses, and tumor metastasis. Studies have demonstrated that CXCL12 activates CXCR4, and high CXCR4 expression may be a potential risk for CRC recurrence or liver metastasis. It has also been associated with poor prognosis [28, 32]. Additionally, CXCR4 expression, after phosphorylation, has been found to have prognostic benefits in CRC [33]. Antonella et al found that CXCL12 could drive the migration of CXCR4-positive cancer cells and macrophages, and could facilitate the molecular crosstalk between them. Macrophages accelerate cancer growth through a CXCL12-potentiated GM-CSF/HB-EGF paracrine loop, leading to poor prognosis and shorter survival time in CRC patients [34]. On the other hand, S1PR1 has been linked to some autoimmune diseases, with Qi et al reporting that aberrant co-expression of S1PR1, as well as signal transducer and activator of transcription 3 (STAT3) are involved in metachronous liver metastasis and poor prognosis in CRC. Mutual activation loop between them enhances proliferation, liver metastasis and invasiveness of CRC cells *in vitro* and *in vivo*, leading to poor prognosis [35]. These findings provide a new perspective to interrogate the prognostic role of differentially expressed immunity genes in CRC through identification of their receptors. Among the 10 immune genes of prognostic value, used to build our model, Fatty Acid Binding Protein 4 (FABP4) [36, 37], NGF [38, 39], Resistin Like Beta (RETNLB) [40, 41], Urocortin (UCN) [42, 43], Vasoactive Intestinal Peptide (VIP) [44, 45], Nerve Growth Factor Receptor (NGFR) [46, 47] have been implicated in direct or indirect involvement in regulation of CRC development and metastasis through other pathways. However, IGKV1-33, IGKV2D-40, IGLV6-57, and OXTR have not been previously associated with CRC. Further analysis is, therefore, required to explore the links between these genes and pathways and CRC to guide the diagnosis and treatment of CRC.

We used the IRGs to further design a simple protocol for monitoring immune status and identifying the clinical outcomes in CRC patients. Clinical risk stratification, based on overall survival time, is important for monitoring the survival of CRC patients. In addition, IRGPI can be used, not only as a prognostic indicator but also, as an indicator of immune status. The immune prognostic indicators developed in this study were based on differential expression of 10 IRGs in CRC, and showed good clinical application. Furthermore, ROC results indicated that our measures were highly accurate. IRGPI showed a considerably high prognostic performance and was associated with age, pathologic tumor stage, TNM stage, lymphatic and vascular invasion, and tumor burden. This prognostic model can be implemented in treatment plans based on the level of immune cell infiltration. Previous studies have reported that CD4 + T and CD8 + T lymphocytes recognize cancer antigens and can be incorporated into immunotherapy against cancer [48–51]. Tumor-associated macrophages (TAM) often contribute to disease progression and counter therapies by providing trophic support to malignant cells. However, they can also mediate antitumor effects and are good targets for incorporation into anticancer therapies in humans through ablation or re-differentiation [52, 53]. On the other hand, neutrophils play a role in tumor initiation, growth, and proliferation, hence can be used as clinical biomarkers and therapeutic targets [54, 55]. Our analysis showed a significant positive correlation between IRGPI with the infiltration of CD4+ T, CD8+ T, dendritic cells, as well as macrophages, and neutrophil. Characterization of the immune permeation landscape is necessary to provide an understanding of tumor-immune interactions. We therefore explored the relationship between IRGPI and immune cell infiltration in CRC, and found a significant positive association between IRGPI and the five levels of immune cell infiltration mentioned above. This suggests a higher degree of infiltration of these immune cells in high-risk patients. Our results further confirm and expand our knowledge into the tumor-related functions of immune cells, as they regulate progression of CRC. Immunotherapy, in which various immune cells play different roles, is the latest revolution in cancer treatment [56].

Inflammation and composition of the tumor microenvironment plays important roles in cancer progression, with numerous studies demonstrating the high school granulocyte to lymphocyte ratio (NLR) as a poor prognostic indicator for several solid malignancies [57]. Neutrophils play an important role in immunobiology of CRC, whereas CD8 + T lymphocyte infiltration is associated with improved survival. Furthermore, neutrophils frequently colocalize with CD8 + T cells and neutrophils and enhance CD8 + T cell responsiveness to T cell receptor triggering. Thus, neutrophils may effectively promote anti-tumor immunity [58]. Tsadik et al, while using a constructed mouse model of human CRC, demonstrated that multifunctional CD4 + effector cells generated after treatment with CD4 + T cell-based adoptive immunotherapy could radically alter tumor metabolism, resulting in disintegration of major antioxidant defense systems and excessive accumulation of ROS in tumor cells. Neutrophils also act synergistically with tumor necrosis factor-α (TNF-α) to enhance oxidative stress and tumor cell death by. This regimen resulted to curing mice diagnosed with CRC [59]. Other studies have reported that interleukin 22 (IL-22), produced by CD4 (+) T cells in CRC tissues, promote activation of transcription factor STAT3 and the expression of DOT1 Like Histone Lysine Methyltransferase (DOT1L). This, in turn, induces expression of core stem cell genes NANOG, SOX2 and POU5F1, leading to increased cancer stemness and tumorigenic potential. In addition, high DOT1L expression in CRC was found to be a poor prognostic factor [60]. CD8 (+) T cell infiltration density in tumors is related to the growth, stage, and metastasis of CRC. Approximately 60% of patients with high-density CD8 (+) cytotoxic T lymphocyte infiltration exhibit Tis/T1 tumors, while those with low density show no such early conditions. In patients with tumor recurrences, fewer CD8 cells were detected regardless of the T stage. However, T stage is negatively related to the number of CD8 infiltration in those without relapse [61]. Dendritic cells (DCs) play a key role in recognizing tumor antigens and inducing T cell-elicited anticancer responses. These cells have been used in development of therapeutic vaccines. CRC cells expressing anti-inflammatory cytokines, such as IL-10 and TGF-β, can influence DC phenotype and enhance tumor escape from immune surveillance. Tumor-associated DCs, therefore, exhibit a number of defects in antigen presentation capacity and altered expression patterns of immune costimulatory molecules in response to immunomodulatory phenotypes. However, DCs in combination with other agents, can also inhibit development and metastasis of CRC [62, 63]. Although immune cells potentially play multiple roles in tumor development, the specific mechanisms in CRC are still not well understood. Th preliminary investigation, presented herein, provides a perspective and foundational knowledge for further studies.

Despite the convincing nature of our findings, some limitations arose and need to be appropriately addressed when interpreting our results. First, transcriptomic analysis can only reflect certain aspects of the immune status, not global alterations. Secondly, a lack of independent cohort validation could have affected the findings. Thirdly, we did not perform any experiments to validate our findings. Many questions remain to be answered for better application of immunotherapy in CRC treatment. For example, further studies are required to elucidate the relationship with regards to metabolomics, proteomics, and immunogenomics and their involvement in CRC. Moreover, the association between immunogenomic disorders and precancerous lesions and CRC development or metastasis needs to be further explored.

In conclusion, we systematically analyzed the role of IRGs in monitoring initiation and prognosis of CRC. Our findings provide new insights that can guide a more detailed phenotyping of immune cells in subsequent clinical trials. This is likely to employ the use of large-scale flow cytometry, in combination with more novel and sensitive sequencing technologies, and will take into account factors the unique microenvironment as well as gut microorganisms. The prognostic signature designed herein may have important clinical implications in the diagnosis, design and development of new therapeutic approaches for CRC. It can be widely used in daily clinical practice. In future, validation of these biomarkers will guide development of novel and effective immunotherapies for CRC patients.

## MATERIALS AND METHODS

### Method summary

We downloaded TCGA data and performed differential analysis to obtain differentially expressed genes. The ImmPort tool was used to identify differentially expressed immune related genes (IRGs). The Database for Annotation, Visualization and Integrated Discovery (DAVID) was employed to perform GO and KEGG enrichment analyses for the differentially expressed IRGs. The STRING database was used to construct a protein interaction network (PPI) for the differentially expressed IRGs. The CBioportal tool was used to create co-expression networks and mutation analysis for differentially expressed IRGs. In addition, we performed gene set enrichment analysis for the differentially expressed IRGs. Differentially expressed TFs in samples from CRC patients were identified, and the transcription factor-immune gene regulatory network was constructed. Finally, independent prognostic analysis and immune cell correlation analysis were performed to construct IRGPI and determine the relationship between immune cell infiltration and IRGPI.

### Data collection and clinical specimen

The Cancer Genome Atlas Program (TCGA: https://cancergenome.nih.gov/), contains large and standardized clinical data for many types of cancer. It is therefore used for high-throughput genomic profiling techniques to study the mechanisms of cancers. From this database, we downloaded the transcriptome profiling data of all CRC samples (including 398 CRC tumor samples and 39 normal control samples) and the corresponding clinical information. This information was screened and used to analyze the survival time, survival status, tumor stage, metastasis status and age after exclusion of incomplete dat. ImmPort is a platform that accurately and timely provides immunological data, including IRGs for cancer research. From this website, we downloaded the IRGs that have been validated to be involved in immunity for further analysis [64].

### Differential gene analysis

The transcriptome data was initially collated and normalized. Next, the limma package in R software was used to perform differential gene analysis using the log2 | fold change | > 1 and the false discovery rate (FDR) < 0.05 as the cutoff values by to obtain a list of significantly differentially expressed genes in the expression matrix. Volcano plots were created using the ggplot2 package [65], and differential gene expression heatmaps were drawn after homogenization of values using the pheatmap package. Finally, differentially expressed immune genes (IRGs) were extracted from DEGs.

### Differential immune gene analysis

The Database for Annotation, Visualization and Integrated Discovery (DAVID) v6.8 (https://david.ncifcrf.gov) is a comprehensive bioinformatics database that integrates a large amount of biological data and practical analysis tools for functional annotation of large-scale protein or genes [66, 67]. This database was used for GO and KEGG enrichment analyses to identity differentially expressed immune genes, and GO and KEGG bubble plots were drawn with the ggplot2 package of R software. The clinical information in the expression profile file data was extracted and used to identify clinically relevant immune genes for correlation analysis with the survival package in R software. The most significant genes were used to draw a forest map. The clinically relevant immune genes were subjected to GO and KEGG analysis in the DAVID database as previously indicated. STRING (Version: 11.0 https://string-db.org/) database integrates and scores the interactions among proteins using computational predictions. It also helps to visualize the intrinsic links among immune genes [68]. After construction of a PPI network of the differentially IRGs, the Cytoscape (v 3.7.0) [69] software was used visualize the interactions. The GO pathway networks of these immune genes were drawn using the BiNGO plugin [70]. CBioportal is a novel model based on TCGA and Gene Expression Omnibus (GEO https://www.ncbi.nlm.nih.gov/geo/), and other large sample cancer genome projects, and provides visualization tools to study and analyze cancer gene data such as expression profiles and clinical prognostic correlations [71, 72]. Based on co-expression and mutation analyses of differential immune genes in this database, the genes with high mutation rate and interacting genes were screened for further analysis.

### Gene set enrichment analysis

Gene expression data of CRC was imported into GSEA 4.0.1 (http://software.broadinstitute.org/gsea/index.jsp), and then analyzed by omics predictions. The pathways and molecular mechanisms associated with them were then further explored. The Hallmark with KEGG gene sets summarizes and represents specific well-defined biological states or processes that, through computational methods, show coherent expression. Each analysis process was performed 1,000 times using the weighted enrichment statistics method. Enriched gene sets with family-wise error rate (FWR) < 0.05 and false discovery rate (FDR) < 0.25 were considered statistically significant.

### TF analysis and TF-IRGs regulatory network

Cistrome Cancer (http://cistrome.org/CistromeCancer/), a comprehensive database of expression profiles and public ChIP-seq profiles from TCGA, predicts target genes and enhancer profiles of TF in TCGA cancer types [73]. Validated transcription factors were downloaded (318 in total, P < 0.05) with statistical relevance to the tumor. These data were combined and the differentially expressed TF were used to draw expression heat map and volcano map. A correlation test was performed based on the TF, combined with prognosis-related immune genes, and the cor cutoff criterion was set to 0.4, and the p-value screening criterion was set to 0.001. These data were used to construct a transcription factor-immune gene regulatory network. The data were mapped with Cytoscape, and the most correlated modules were selected using the MCODE plugin [74].

### Independent prognostic analysis and evaluation

We collected clinical information of 398 patients. A total of 60 patients who did not survive beyond 3 months and whose clinical information was incomplete were excluded to reduce the interference of unrelated factors. A clinical correlation analysis was then performed on 338 patients. After analyzing the correlation between the clinically relevant immune genes and each clinical trait, univariate and multivariate independent prognostic forest plots were constructed for the immune genes and clinical traits using the survival package under the condition that only the effect of a single trait on survival and the effect of multiple factors on survival were considered comprehensively. In reference to the median prognostic index (PI), patients were grouped to high- and low-risk groups. Similarly, survival models were constructed with the survival package of R software based on the grouping files of PI values to assess the performance of PI on different subtypes of CRC. Multivariate analysis was performed based on IRG, and integrated IRG was used as an independent prognostic indicator to create IRGPI. Subsequently, risk curves for the high and low-risk groups were plotted using the pheatmap package. IRGPI was constructed by summing the expression of the central immunity genes with the multiplication Cox regression coefficient. To assess the validity of the model, group files were calculated and ROC curves were plotted with the survivalROC package.

### Immune cell correlation analysis

Tumor Immune Estimation Resource (TIMER https://cistrome.shinyapps.io/timer/), is a comprehensive database containing immune cell infiltration data for various tumors. The infiltration level of six immune cells (B cells, CD4 + T cells, CD8 + T cells, Neutrophils, Macrophages and Dendritic cells) was obtained from the TCGA and other publicly validated databases containing tumor information. This platform was used in this study [75, 76]. Data on immune cell infiltration in tumors was downloaded from TIMER, typed them jointly with central immune gene and risk value files. These data were used to construct correlation graphs for the relationship between immune cells and IRGPI, P < 0.05 was considered statistically significant.

### Statistical analysis

The data were processed, analyzed, and presented using the R software and its associated software packages. Our research results were complemented and validated with practical and accurate databases. The performance of the prognostic index was evaluated using the area under curve (AUC) value of the survival ROC curve [77]. Clinical phenotypes were compared using independent t-tests. p < 0.05 was considered statistically significant.
